# The role of fibromodulin in myocardial fibrosis in a diabetic cardiomyopathy rat model

**DOI:** 10.1002/2211-5463.13935

**Published:** 2024-11-26

**Authors:** Xiyan Dai, Fan Yang, Dongping Chen, Lu Yang, Zhihui Dong, Can Chen, Jianmin Xiao

**Affiliations:** ^1^ Binhaiwan Central Hospital of Dongguan China; ^2^ Maoming People's Hospital China; ^3^ The First Clinical Medical College Jinan University Guangzhou China

**Keywords:** diabetic cardiomyopathy, fibromodulin, myocardial fibrosis

## Abstract

Diabetic cardiomyopathy (DCM) is pathologically characterized by excessive deposition of extracellular matrix proteins, leading to myocardial fibrosis. Fibromodulin (Fmod) plays a crucial role in the pathogenesis of fibrotic diseases. However, the role and mechanism of Fmod in DCM‐related myocardial fibrosis remain unclear. In the present study, we established a DCM rat model and an *in vitro* model of rat primary cardiac fibroblasts (RPCFs) exposed to high glucose. We assessed mRNA and protein expression levels of Col1a1, Col3a1, α‐SMA and Fmod in both models. *Fmod*‐overexpressing (ov‐Fmod) and *Fmod*‐knockdown (si‐Fmod) rat cardiac fibroblasts (RCFs) were generated. Subsequently, whole RNA sequencing was conducted on ov‐Fmod RCFs. The gene *Col15a1* was evaluated in the DCM rat and all cell models. The correlation between plasma levels of Fmod and Col15a1 in DCM rat models was assessed. Transcription and protein levels of Fmod, Col1a1, Col3a1 and α‐SMA were significantly elevated in DCM rat hearts and RPCFs. In ov‐Fmod RCFs, fibrosis markers were similarly increased, except for Col3a1, which decreased. The Col1a1/Col3a1 ratio was elevated. Conversely, knocking down Fmod yielded opposite results. Gene Ontology and Kyoto Encyclopedia of Genes and Genomes analyses indicated that Fmod participates in multiple fibrosis‐related pathways, affecting Col15a1. Expression of *Col15a1* was significantly decreased in all models, compared to controls, except in si‐Fmod RCFs. Importantly, Col15a1 and Fmod in plasma exhibited an inverse relationship in DCM. In summary, Fmod is implicated in DCM, with *Fmod* overexpression downregulating Col15a1 and increasing the Col1a1/Col3a1 ratio. This mechanism may influence diastolic heart failure in DCM by modulating myocardial stiffness and elasticity.

## Graphical abstract

Fibromodulin (Fmod) levels were higher in heart tissue of a diabetic cardiomyopathy (DCM) rat model, and in rat primary cardiac fibroblasts induced by high glucose, compared to the control group. *Fmod*‐overexpressing rat cardiac fibroblasts had decreased Col15a1 levels and an increased Col1a1/Col3a1 ratio. Conversely, knocking down *Fmod* yielded opposite results.

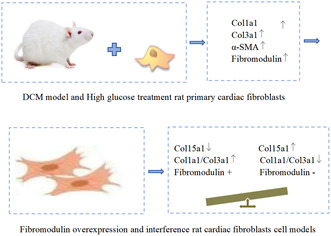

**Abbreviations**
Acta2: actin alpha 2, smooth muscle
Col15a1: collagen type XV alpha 1 chain
Col1a1: collagen type I alpha 1 chain
Col3a1: collagen type III alpha 1 chain
DCM: diabetic cardiomyopathy
DEG: differently expressed gene
DMEM: Dulbecco's modified Eagle's medium
ECM: extracellular matrix
Fmod: fibromodulin
FPKM: fragments per kilobase of exon model per million mapped fragments
GO: Gene Ontology
HE: hematoxylin and eosin
HG group: high glucose group
KEGG: Kyoto Encyclopedia of Genes and Genomes
MOD: mean optical density
NG group: normal glucose control group
OC group: osmotic control group
ov‐Fmod: overexpression‐Fmod
PBS: phosphate‐buffered saline
RCFs: rat cardiac fibroblasts
RPCFs: rat primary cardiac fibroblasts
RT‐qPCR: reverse transcriptase‐quantitative PCR
si‐Fmod: small interfering RNA interference‐Fmod
siRNA: small interfering RNA
STZ: streptozotocin
α‐SMA: α‐smooth muscle actin

Diabetic cardiomyopathy (DCM), a significant complication of diabetes mellitus, was initially described by Rubler *et al*. [1]. It represents a distinct form of cardiomyopathy occurring in diabetic patients, independent of concurrent coronary artery disease, hypertension or valvular heart disease [2, 3]. However, the exact pathogenesis of myocardial fibrosis in DCM remains unclear. Fibromodulin (Fmod), classified as a small leucine‐rich proteoglycan within the extracellular matrix (ECM), plays a crucial role in regulating matrix composition by interacting with various collagen proteins. This regulatory function extends to cellular processes such as proliferation, differentiation, and adhesion, thereby influencing ECM formation and maintaining tissue integrity [4]. Fmod is involved in diverse tissue development and regeneration, including cartilage, bone, tendon, muscle and skin [5]. Although the role of Fmod in cardiovascular diseases is rarely reported, its specific mechanisms in DCM‐related myocardial fibrosis remain unexplored. Therefore, the present study aimed to investigate the role of Fmod in DCM‐related myocardial fibrosis. Insights gained from this research could reveal novel biological markers and therapeutic targets for managing myocardial fibrosis in DCM. [Correction added on 28 January 2025 after first online publication: The word “Fibronectin (Fmod)” has been replaced with “Fibromodulin (Fmod).”]

## Materials and methods

### Establishment of diabetic rat model

A detailed description of the DCM rat model was provided in our previous reports [6, 7]. The rats were provided by the SPF Biotechnology company (SPF, Beijing, China) and housed in a sanitary animal facility under a 12 : 12 h light/dark photocycle at 20–25°C and 40–70% relative humidity. In brief, male Sprague–Dawley (SD) rats aged 8 weeks were randomly assigned to either the normal control group (Control, *n* = 5) or the DCM model group (DCM, *n* = 5). Rats in the DCM group received intraperitoneal injections of 1% streptozotocin (STZ) (#S817944; Macklin, Shanghai, China; 50 mg·kg^−1^). One week after injection, blood glucose levels were measured from the tail vein, with a random glucose level > 16.7 mmol·L^−1^ indicating elevated blood glucose. Echocardiographic data were collected and analyzed 20 weeks post‐injection to assess cardiac function, performed by an experienced ultrasound physician using an L15‐7io probe (ultrasound transducer; Sonic Concepts, Philips, Bothell, WA, USA). Care was taken to minimize discomfort, distress and pain to the animals. Following the blood samples and cardiac tissue collection, all the animals were killed by an overdose of anesthesia. All animal experiments were conducted in accordance with the ‘Guide for the Care and Use of Laboratory Animals’ published by the National Academy of Sciences. Approval for all procedures was obtained from the Laboratory Animal Ethics Committee of Ji'nan University (Approval No. 20210826‐29).

### Histological analysis and immunohistochemistry analysis

Upon euthanasia under anesthesia with an intraperitoneal injection of phenobarbital sodium (60–80 mg·kg^−1^, H20057384; Fujian Mindong Rejuenation, Fujian, China). All rats' hearts were excised, rinsed in sterile saline, blotted dry and promptly fixed in 4% paraformaldehyde. Subsequently, tissues were dehydrated, cleared, immersed in wax, embedded into paraffin blocks and sectioned into 3‐μm thick slices. Myocardial architecture and arrangement were evaluated using hematoxylin and eosin (HE) staining (#G1120; Soleberg, Solarbio, Beijing, China), whereas extent of collagen fibrosis was assessed with Masson's trichrome staining (#BA4079B; BaSO, Wuhan, China). For immunohistochemistry, the following antibodies were used in accordance with the manufacturer's instructions: anti‐α‐smooth muscle actin (α‐SMA; #14395‐1‐AP; Proteintech, Rosemont, IL, USA; dilution 1 : 4000), anti‐collagen type I alpha 1 chain (Col1a1; Collagen I; #14695‐1‐AP; Proteintech; dilution 1 : 500), anti‐collagen type III alpha 1 chain (Col3a1; Collagen III; #22734‐1‐AP; Proteintech; dilution 1 : 1500). Histopathology and immunohistochemistry procedures were conducted by a technician with 20 years of experience. All subsequent analyses were performed by staff who were blinded to the experimental groups. image‐pro plus, version 6.0 (Media Cybernetics, Rockville, MD, USA) was used to quantify the brown stained areas in each image, with mean optical density (MOD) calculated as integrated optical density (sum)/area (sum).

### Isolation, immunofluorescence identification and culture of rat primary cardiac fibroblasts (RPCFs)

RPCFs were isolated from neonatal SD rats aged 1–3 days. Following death of the suckling rats, ventricular tissues were isolated and immersed in pre‐cooled D‐Hanks solution (#BL559A; Biosharp, Hefei, China) at 4 °C. The heart tissues were then cut into 1 mm^3^ pieces using ophthalmic scissors and digested with 0.075% trypsin (#25200072, Gibco, Grand Island, USA) at 4 °C for 16 h. After trypsin digestion, the tissues were further treated with 0.05% collagenase type II (#C8150; Solarbio, Beijing, China) at 37 °C for 60 min. Cell suspensions were filtered through a cell sieve and then seeded in complete Dulbecco's modified Eagle's medium (DMEM) medium (containing 5.5 mmol·L^−1^ glucose, 10% fetal bovine serum and 1% penicillin–streptomycin). After 90 min, the supernatant was removed, and the cells were cultured in complete medium with fetal bovine serum reduced to 2%. Cells were seeded in six‐well plates (2 × 10^5^ cells per well) with cell‐climbing coverslips and fixed overnight in 4% paraformaldehyde. The cells were incubated with Vimentin Monoclonal antibody (#60330‐1‐Ig; Proteintech; dilution 1 : 500) overnight at 4 °C on a shaker. After the primary antibody was washed away with phopshate‐buffered saline (PBS), the cells were incubated with CoraLite488‐conjugated goat anti‐mouse IgG(H+L) (#SA00013‐1; Proteintech) on a shaker, protected from light for 40 min. The secondary antibody was washed away with PBS, and a drop of 4′,6‐diamidino‐2‐phenylindole was added to the cells before they were covered with a coverslip and incubated in the dark for 30 min. The membrane was incubated for 40 min on a shaker, protected from light with PBS, covered with a coverslip and incubated in the dark for 30 min. The cultured fibroblasts were divided into three groups: normal control group (5.5 mmol·L^−1^ glucose, NG group), high glucose group (30 mmol·L^−1^ glucose, HG group) and osmotic control group (5.5 mmol·L^−1^ glucose and 24.5 mmol·L^−1^ mannitol, OC group).

### Culture of rat cardiac fibroblasts (RCFs)

RCFs (#R6300; ScienCell, Carlsbad, CA, USA) were cultured at 37 °C in a 5% CO_2_ incubator. The medium was changed every 3 days, and subculture was performed when the culture reached 90–95% confluence.

### Vector construction and RCFs transfection

For pLVX‐Fmod‐puro plasmid construction, the rat Fmod CDS fragment was cloned into the pLVX‐puro vector (Jieri, Shanghai, China). HEK‐293T cells were co‐transfected with the pLVX‐Fmod‐puro construct, psPAX2 and pMD2.G in a ratio of 4 : 3 : 1 to produce lentiviral particles. The pLVX‐puro empty vector served as a control. Lentiviruses were harvested 48 h after transfection. Stable cell lines were established by infecting RCFs with lentiviral particles followed by selection with puromycin. RCFs were cultured in DMEM medium (containing 5.5 mmol·L^−1^ glucose). The Fmod‐overexpressing group (ov‐Fmod group) and vector control group (ov‐vector) were cultured in complete medium with 2% fetal bovine serum and 4 μg·mL^−1^ puromycin. Upon reaching 80% confluency or greater, cells were passaged, and second‐generation cells were selected for subsequent experiments. The efficiency of Fmod overexpression was evaluated by western blot analysis and reverse transcriptase‐quantitative PCR (RT‐qPCR) 48 h after lentiviral transfection.

### Transfection of small interfering RNA (siRNA)

siRNA was transfected into RCFs using a transient transfection reagent (#AD600075; ZETA Life, Menlo Park, CA, USA) when cells reached 30–50% confluence. siRNAs were obtained from IGE Biotechnology (Guangzhou, China) and dissolved in diethyl pyrocarbonate‐treated water to a final concentration of 20 μm. The siRNAs were combined with the siRNA transfection reagent at a 1 : 1 volume ratio in accordance with the manufacturer's recommendations and incubated for 15 min at room temperature. Cells were then incubated with 2 mL of DMEM (5% fetal bovine serum) containing 16 μL of the transient transfection complex without penicillin–streptomycin. After 24 h, the medium was replaced with DMEM containing 5% fetal bovine serum and 1% penicillin–streptomycin. RNA and protein extraction was performed 48 h after transfection for subsequent experiments. The si‐Fmod and si‐NC sequences are listed in Table S1.

### Whole RNA sequence of CFs cells overexpressing Fmod and bioinformatics analysis

Total RNA was extracted from transfected cells using TRIzol reagent in accordance with the manufacturer's instructions. RNA purity and concentration were assessed using a NanoDrop 2000 spectrophotometer (Thermo Fisher Scientific, Waltham, MA, USA), whereas RNA integrity was evaluated with an Agilent 2100 Bioanalyzer (Agilent Technologies, Santa Clara, CA, USA). Transcriptome libraries were prepared in accordance with the manufacturer's instructions, and sequencing and analysis were conducted by Ouyi Biotechnology (Shanghai, China). Sequencing was performed on the Illumina Novaseq 6000 platform (Illumina Corp., San Diego, CA, USA), generating 150 bp paired‐end reads. Approximately 49 million raw reads were obtained per sample. Raw reads in FASTQ format were processed using the fastp (HaploX Biotechnology, Shenzhen, China) to obtain clean reads by filtering out low‐quality reads, which were subsequently used for data analysis. Alignment to the reference genome was performed using hisat2 (Johns Hopkins University, Baltimore, MD, USA), and gene expression levels were quantified as fragments per kilobase of transcript per million mapped reads (FPKM). The counts of reads for each gene were obtained using htseq‐count (Genome Biology Unit, European Molecular Biology Laboratory, Heidelberg, Germany). Differentially expressed genes (DEGs) were identified using deseq2 (https://bioconductor.org/packages/release/bioc/html/DESeq2.html), with genes meeting thresholds of *q* < 0.05 and fold change > 2 or fold change < 0.5 considered as DEGs. Hierarchical clustering analysis of DEGs was conducted using r, version 3.2.0 (R Foundation, Vienna, Austria) to visualize gene expression patterns across groups and samples. Gene Ontology GO) (http://geneontology.org) and Kyoto Encyclopedia of Genes and Genomes (KEGG) (https://www.genome.jp/kegg) enrichment analyses of DEGs were performed using hypergeometric distribution algorithms to identify significantly enriched functional terms. Bar charts and bubble diagrams illustrating significantly enriched functional entries were generated using r, version 3.2.0.

### RT‐qPCR

Total RNA was extracted from rat myocardium, RPCFs and RCFs using Trizol reagent, followed by reverse transcription to cDNA using the PrimeScript™ FAST RT reagent Kit with gDNA Eraser kit (#RR092A; Takara, Shiga, Japan). Target genes were amplified by qPCR using a real‐time fluorescence PCR instrument (#SLAN‐96P; Hongshi, Shanghai, China) and Universal SYBR qPCR Master Mix (#BL697A; Biosharp) in accordance with the manufacturer's instructions. Tubb5 was used as an internal reference gene for mRNA normalization. The PCR primer sequences are listed in Table S2.

### Western blot analysis

The expression levels of target proteins were analyzed by western blotting following standard protocols. Proteins were separated on 6–10% polyacrylamide gradient gels and transferred to poly(vinylidene difluoride) membranes (#ISEQ00010; Millipore, Burlington, MA, USA). The following primary antibodies and concentrations used were: Fmod (#13281‐1‐AP; Proteintech; dilution 1 : 500), COL1A1 (Collagen I; #14695‐1‐AP; Proteintech; dilution 1 : 2000), COL3A1 (Collagen III; #22734‐1‐AP; Proteintech; dilution 1 : 1000), α‐SMA (#14395‐1‐AP; Proteintech; dilution 1 : 5000). HRP‐coupled goat anti‐rabbit IgG secondary antibody (#ab205718; Abcam, Cambridge, UK; dilution1 : 50 000) and goat anti‐mouse secondary antibody (#FmodM007; FUDE, Hangzhou, China; dilution1 : 25 000) were used for detection. β‐tubulin (#Fmod0064; FUDE; dilution 1 : 5000) served as the internal reference protein.

### Enzyme‐linked immunosorbent assay (ELISA)

The levels of Fmod and Col15a1 released in plasma from DCM rat models and cell supernatants were quantified using ELISA kits (#MM‐70379R2, #MM‐72180R1‐96T; Meimian, Jiangsu, China) in accordance with the manufacturer's instructions.

### Statistical analysis

All data were subjected to statistical analysis using prism, version 8.0 (GraphPad Software Inc., San Diego, CA, USA). Differences between two groups were evaluated using a two‐tailed Student's *t*‐test. Statistical comparisons among multiple groups were performed using one‐way ANOVA. The Mann–Whitney non‐parametric test was applied for data that did not conform to normal distribution. Pearson correlation coefficients were calculated to assess correlations. *P* < 0.05 was considered statistically significant.

## Results

### Establishment of diabetic rat model

The DCM animal model was successfully established. Compared to the control group, rats in the model group exhibited elevated blood glucose levels (> 16.7 mmol·L^–1^), echocardiographic evidence of enlarged hearts and decreased EF values indicative of impaired cardiac function. These results confirm the successful construction of the DCM model, with detailed data previously reported [7].

### Fmod increased in DCM rat heart and RPCFs treated with high glucose of fibrosis

**Fig. 1:**
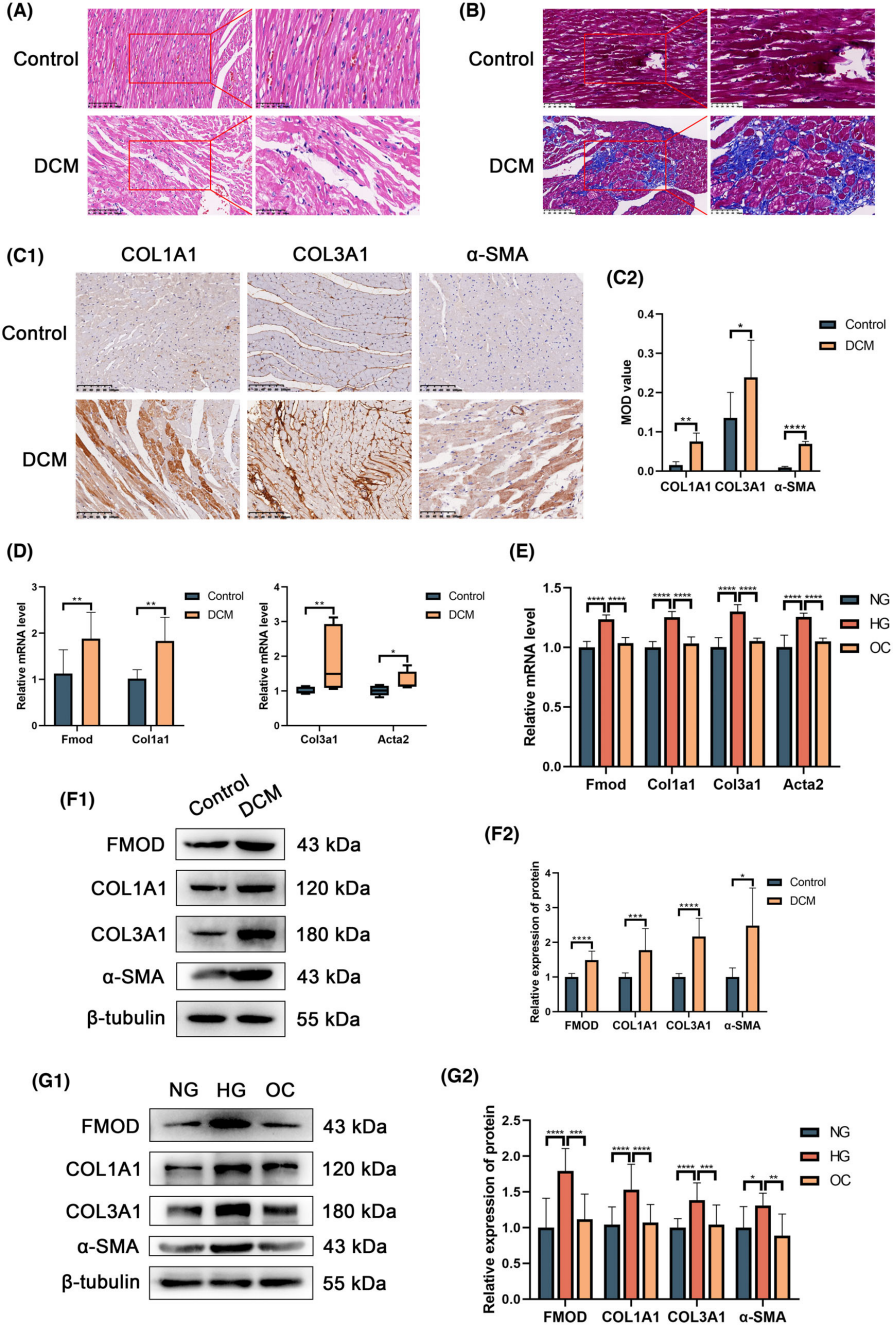
Fibrosis indicators and Fmod increased in DCM rat hearts and RPCFs treated with high glucose. (A) HE staining results. (B) Masson staining stained the collagen fiber blue, the muscle fiber cytoplasm red and the nucleus brown. Note the irregular arrangement of myocardial fibers and more blue staining in the DCM group. (C1, C2) Immunohistochemistry analysis. Brown indicates positive expression of fibrosis indicators. The vertical coordinate represents the MOD value in image pro plus, version 6.0, where higher values represent stronger positive expression. (D, E) RT‐qPCR results of rat hearts and RPCFs, respectively, and the results represent relative mRNA expression levels, which were normalized to the reference gene (Tubb5). (F1, F2; G1, G2) Western blot results of rat hearts and RPCFs, respectively, and the results represent relative protein expression levels, which were normalized to the reference gene (β‐tubulin). (A, left; B, left) Magnification 200×; scale bar = 100 μm. (A, right; B, right) Magnification 400×; scale bar = 50 μm. (C1) Magnification 200×; scale bar = 100 μm. The data are presented as the mean ± SD or median (Q1, Q3). An unpaired *t* test was used with respect to COL3A1 in (C2) and Fmod in (D) (left). Welch's *t* test was used with respect to COL1A1 and α‐SMA in (C2), Col1a1 in (D) (left) and all indicates in (E2). A Mann–Whitney test was used in (D) (right). Ordinary one‐way ANOVA was used with respect to Fmod and Col1a1 in (F) and FMOD, COL1A1 and α‐SMA in (G2). Brown‐Forsythe and Welch ANOVA tests were used with respect to Col3a1 and Acta2 in (F) and COL3A1 in (G2). Compared with the control group: *P < 0.05, **P < 0.01, ***P < 0.001, ****P < 0.0001, *N* = 4 (animal experiment), *N* = 3 (cell experiment).

HE staining revealed a regular arrangement of myocardial fibers and cardiomyocytes in control rat hearts. By contrast, hearts from the DCM group showed irregular arrangements with thickened and fragmented fibers and increased interstitial spacing. Masson trichrome staining demonstrated sparse collagen fibers in the control group, whereas the DCM group exhibited a higher density of collagen fibers in the mesenchymal region. Immunohistochemistry analyses indicated increased expression levels of Col1a1, Col3a1 and α‐SMA in DCM rat hearts. Figure S1 shows that the purity of primary cardiac fibroblasts was more than 90%. RT‐qPCR and western blot analysis results confirmed elevated levels of Fmod, Col1a1, ol3a1 and α‐SMA in both DCM rat hearts and high glucose‐treated RPCFs. These findings suggest significant collagen fiber production in the DCM rat model and its promotion in RPCFs exposed to high glucose *in vitro*, with concurrent increases in Fmod levels (Fig. 1).

### Col1a1, Col3a1, Col1a1/Col3a1 and α‐SMA changed in ov‐Fmod and si‐Fmod RCFs

**Fig. 2:**
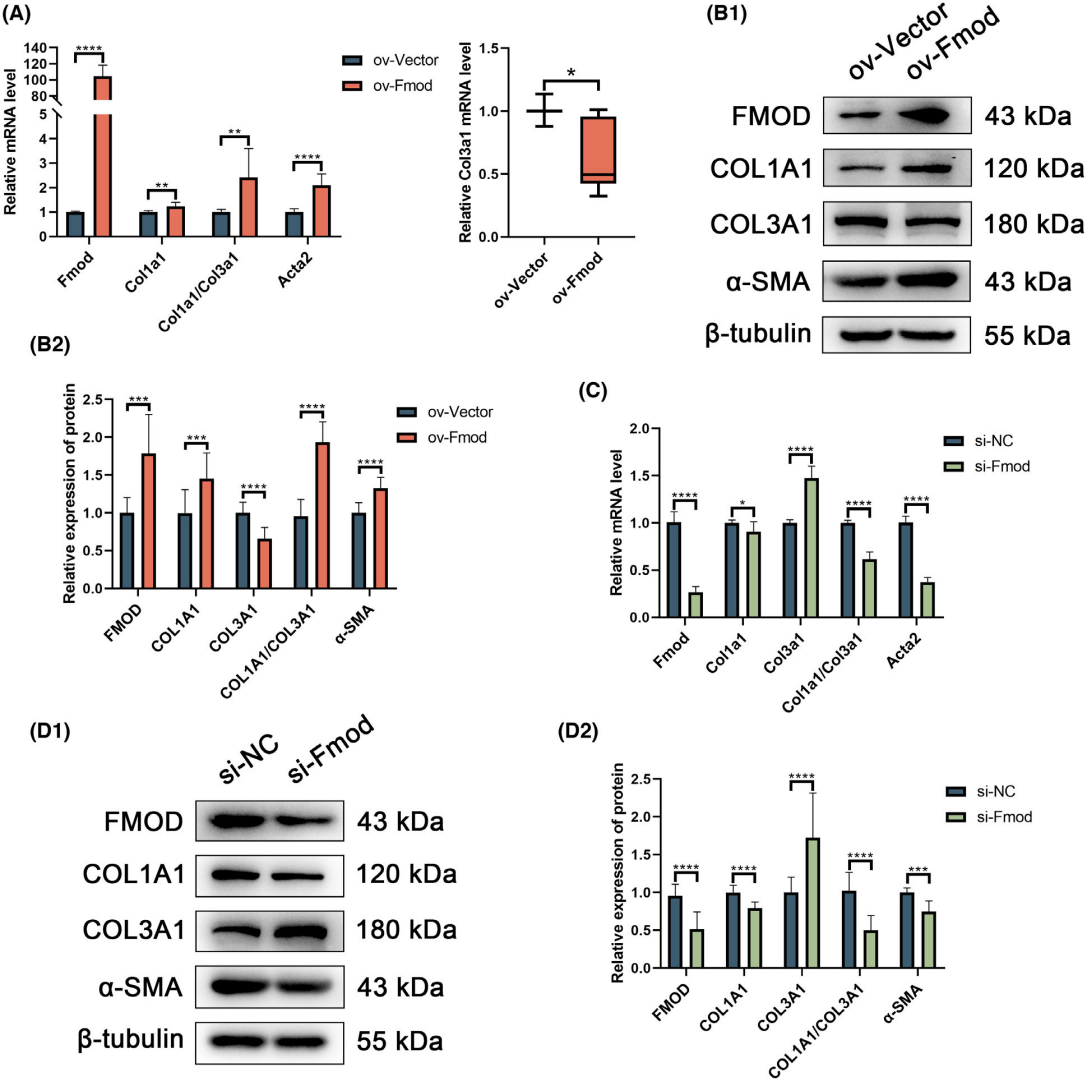
Col1a1, α‐SMA and Col1a1/Col3a1 ratio increased and Col3a1 decreased in ov‐Fmod RCFs, whereas the opposite trend was found for the si‐Fmod RCFs RT‐qPCR (A, C) Col1a1, Col1a1/ Col3a1 and Acta2 increased in the ov‐Fmod group and decreased in the si‐Fmod group, whereas Col3a1 decreased in the ov‐Fmod group and decreased in the si‐Fmod group. Similarly, western blot results (B1, B2, D1 and D2) showed that Col1a1, Col1a1/ Col3a1 and α‐SMA increased in the ov‐Fmod group and decreased in the si‐Fmod group, whereas Col3a1 decreased in the ov‐Fmod group and decreased in the si‐Fmod group. The data are presented as the mean ± SD except max/min in (A) (right). Welch's *t* test was used with respect to all indicators in (A) (left), Fmod in (B2), Fmod, Col1a1, Col3a1 and Col1a1/Col3a1 in (C), and COL3A1 and α‐SMA in (D2). A Mann–Whitney test was used in (A) (right). An unpaired *t* test was used with respect to COL1A1 and COL1A1/COL3A1 in (B2), Acta2 in (C), and FMOD, COL1A1 and COL1A1/COL3A1 in (D2). Compared with Control group: *P < 0.05, **P < 0.01, ***P < 0.001, ****P < 0.0001, *N* = 3.

Compared to the control group, RT‐qPCR and western blot analysis of the ov‐Fmod group showed a significant increase in both mRNA and protein expression levels of Fmod, indicating successful transfection. Conversely, Fmod expression decreased significantly in the si‐Fmod group, confirming effective knockdown of Fmod. Additionally, the transcription and protein levels of Col1a1 and α‐SMA were markedly elevated, whereas Col3a1 expression decreased in the ov‐Fmod group. Consequently, the Col1a1/Col3a1 ratio was significantly higher in ov‐Fmod RCFs. By contrast, si‐Fmod RCFs exhibited opposite trends with reduced Col1a1 and α‐SMA levels, and an elevated Col3a1 expression, resulting in a lower Col1a1/Col3a1 ratio (Fig. 2).

### The ov‐Fmod RCFs transcriptomic results suggest that Col15a1 was involved in the fibrosis‐associated enrichment

**Fig. 3:**
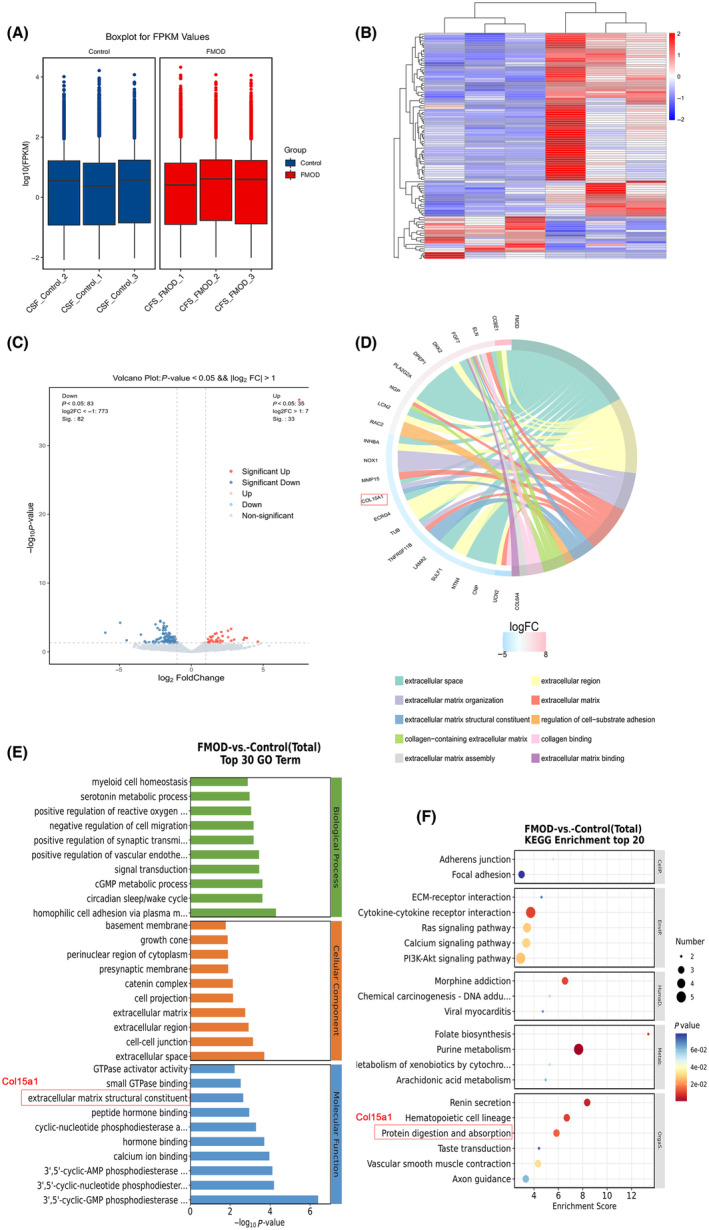
Changes in transcriptome of fibrosis‐related differential genes of Fmod transfected cells six samples of two groups were used in transcriptome analysis (*n* = 3, each group). (A) Boxplot of FPKM values of genes across samples showing consistent levels of gene expression between groups of samples. Boxplots showing five‐number summaries (minimum, first quartile, median, third quartile, maximum). (B) The clustered heatmap shows the DEGs. The red in the graph indicates relatively highly expressed protein‐coding genes and the blue indicates relatively lowly expressed protein‐coding genes. (C) The volcano plot shows the overall distribution of genes in the two groups. Red and blue are the significantly different genes. (D) GO chord plots were constructed to highlight core targets related to fibrosis. (E) Bar graph demonstrating the top30 entries of GO enrichment analysis of DEGs. (F) Bubble plot showing the top20 entries of KEGG enrichment analysis. Col15a1 was marked with red box in (D), (E) or (F).

Box plots illustrating gene expression levels demonstrated consistent overall levels and dispersion of gene expression within and between the control and Fmod overexpression groups of RCF samples (*n* = 3) (Fig. 3A). The hierarchical clustering heatmap revealed significantly different mRNA expression profiles in the Fmod overexpression group compared to the control group (Fig. 3B). Volcano plot analysis identified 118 differentially expressed mRNAs in the Fmod overexpression group, comprising 35 up‐regulated and 83 down‐regulated mRNAs (Fig. 3C). GO chord plots were constructed to highlight core targets related to fibrosis (Fig. 3D). GO enrichment analysis of differentially expressed genes revealed significant enrichment in biological processes such as cell–cell junction (GO:0005911), extracellular matrix organization (GO:0031012) and enzymatic activities including 3′,5′‐cyclic‐GMP phosphodiesterase activity (GO:0047555), 3′,5′‐cyclic‐nucleotide phosphodiesterase activity (GO:0004114) and 3′,5′‐cyclic‐AMP phosphodiesterase activity (GO:0004115) (Fig. 3E). KEGG enrichment analysis showed that differentially expressed genes in the Fmod overexpression group were mainly enriched in pathways such as purine metabolism (rno00230), renin secretion (rno04924) and folate biosynthesis (rno00790) (Fig. 3F). These findings were compared with our previous RNA‐seq data from the DCM rat model. Consequently, Col15a1 was selected for further validation experiments as a result of its consistent down‐regulation in both sequencing datasets. Notably, Col15a1 was implicated in fibrosis‐related processes including extracellular space, extracellular matrix organization, and extracellular matrix structural constituent in GO and KEGG enrichment analyses (Fig. 3).

### Col15a1 decreased in DCM rat model and *in vitro* experiment and was a downstream gene of Fmod

**Fig. 4:**
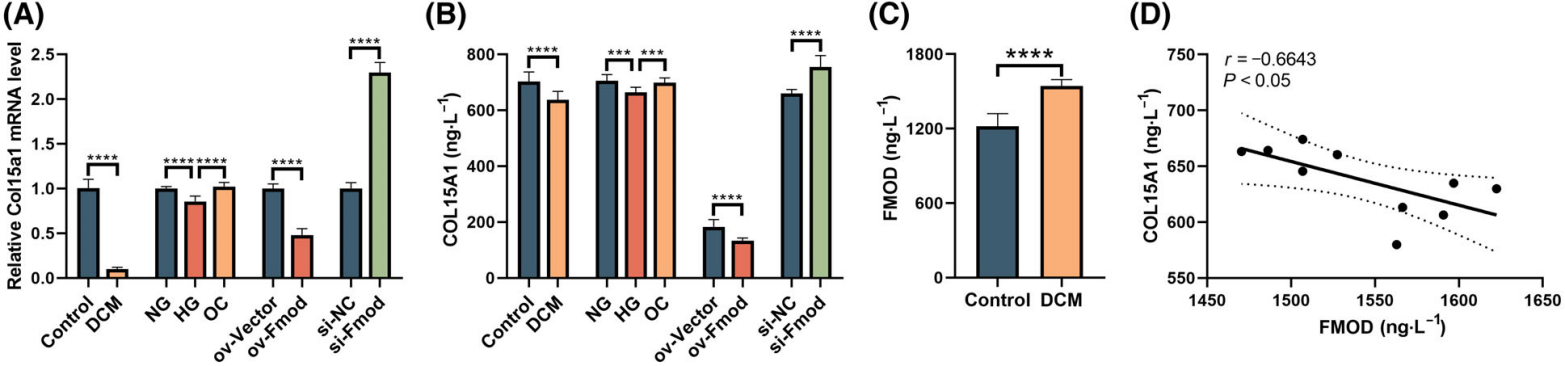
Change of Col15a1 in DCM rat model and *in vitro* experiment and correlation of plasma FMOD and COL15A1 levels in DCM rats. RT‐qPCR results (A) and ELISA results (B) showing that Col15a1 was decreased in the DCM animal model, high glucose‐treated RPCF, and overexpressing‐FMOD RCF. Col15a1 rose in si‐Fmod RCF. ELISA results (C) showing that plasma FMOD levels increased in DCM rats. Correlation analysis results (D) showing a negative correlation between plasma FMOD levels and COL15A1 levels in the diabetic rat model. The data are presented as the mean ± SD. Welch's *t* test was used with respect to DCM in (A), ov‐Fmod and siFmod in (B), and DCM in (C). An unpaired *t* test was used with respect to ov‐Fmod and si‐Fmod in (A) and DCM in (B). Brown‐Forsythe and Welch ANOVA tests were used with respect to NG, HG and OC in (A). Ordinary one‐way ANOVA was used with respect to NG, HG and OC in (B). Pearson correlation coefficients were used in (D). Compared with the control group: **P < 0.01, ***P < 0.001, ****P < 0.0001, *N* = 4 (animal experiment), *N* = 3 (cell experiment).

RT‐qPCR and ELISA analyses demonstrated that Col15a1 levels were decreased in DCM rat hearts and RPCFs treated with high glucose compared to the control group. Subsequently, the relationship between Fmod and Col15a1 was investigated. Correlation analysis revealed a negative correlation between the levels of Fmod and Col15a1 in the plasma of DCM rats. Furthermore, mRNA and protein levels of Col15a1 significantly decreased in Fmod‐overexpressing (ov‐Fmod) RCFs. Conversely, Col15a1 levels increased significantly upon knockdown of Fmod using siRNA. These findings suggest that Fmod may influence myocardial fibrosis by modulating the expression of Co115a1 (Fig. 4).

## Discussion

The present study revealed significant upregulation of Fmod in the STZ‐induced DCM rat model and in cardiac fibroblasts treated with high glucose (HG). Overexpression of Fmod in RCFs altered the Col1a1/Col3a1 ratio and the expression of Col15a1. This study represents the first investigation into the role and potential mechanism of Fmod in DCM‐related myocardial fibrosis, offering novel theoretical insights and potential therapeutic targets for understanding the pathogenesis of diabetic myocardial fibrosis.

Myocardial fibrosis, characterized by enlargement of the cardiac interstitium as a result of the accumulation of ECM proteins, is a common feature in various cardiac pathologies [8]. Persistent metabolic dysregulation in DCM leads to prolonged exposure to fibrotic stimuli, triggering the activation and proliferation of fibroblasts [9]. In our study, we established a rat model of DCM through intraperitoneal injection of STZ and an *in vitro* cell model using high glucose treatment in RPCFs. The conversion of cardiac fibroblasts to myofibroblasts, characterized by α‐SMA expression, is a crucial process in fibrotic pathology [10]. Additionally, Col1a1 and Col3a1 are commonly assessed as markers of cardiac fibrosis [11]. Our results demonstrated fibrotic changes in the rat heart, with increased expression of Col1a1, Col3a1 and α‐SMA observed in both the animal and cell models, consistent with previous findings [12, 13]. Thus, our study underscores the presence of fibrosis in the DCM rat model and *in vitro* cell model.

Fmod belongs to the class II small leucine‐rich proteoglycan family and plays a pivotal role by binding directly to extracellular matrix structural components such as collagen and lysyl oxidase. This interaction regulates collagen cross‐linking, packing, assembly, and fibril architecture through a multivalent mechanism [5]. Dysfunctions in Fmod have been increasingly linked to various diseases including intervertebral disc diseases, osteoporosis, arthritis, joint laxity, caries, vascular diseases, heart failure, gynecological diseases, diabetic nephropathy and fibrotic disorders across multiple organs [5]. It has been implicated in the pathogenesis of dilated cardiomyopathy and hypertrophic cardiomyopathy [14], with further support from findings by Liang *et al*. [15] emphasizing its involvement in cardiovascular diseases. This study marks the first report linking Fmod to DCM, revealing increased Fmod levels in both the hearts of DCM rats and in high glucose‐treated RPCFs. This finding contrasts with previous reports; Andenaes *et al*. [16] suggested that Fmod does not promote cardiac fibroblast migration or myofibroblast transdifferentiation, proposing anti‐fibrotic effects in cultured cardiac fibroblasts. A significant distinction between these studies lies in the context of diabetes exposure. Therefore, we hypothesize that the promotion of fibrosis by Fmod may be specifically associated with diabetic cardiomyopathy, highlighting the need for further investigation.

To investigate the mechanism underlying the role of Fmod in myocardial fibrosis, we overexpressed Fmod in RCFs and assessed fibrosis‐related markers. Our findings revealed that Fmod overexpression led to increased levels of α‐SMA and collagen fibers in RCFs. Interestingly, we observed a reduction in Col3a1 expression, which contrasts with previous findings and suggests complex regulatory interactions in tissues and cells exposed to high glucose stimulation. Importantly, our study demonstrated that Fmod overexpression in RCFs elevated the Col1a1/Col3a1 ratio, which may better elucidate how Fmod influences myocardial fibrosis. In cardiac extracellular matrix, Col1a1 primarily determines myocardial stiffness, whereas Col3a1 influences myocardial elasticity [17]. An elevated Col1a1/Col3a1 ratio has been implicated in diastolic heart failure in DCM. Numerous studies have highlighted the association between an elevated Col1a1/Col3a1 ratio and myocardial fibrosis. For example, in mice with myocardial fibrosis post‐myocardial infarction, Col1a1 levels were significantly increased, whereas Col3a1 levels decreased, resulting in an elevated Col1a1/Col3a1 ratio [18]. miR‐30a has been shown to attenuate cardiac fibrosis in myocardial infarction rats by reducing the Col1a1/Col3a1 ratio [19]. Similarly, in DCM rat myocardium, the Col1a1/Col3a1 ratio was notably elevated [20]. Combining these findings with our study results, it is suggested that Fmod may contribute to myocardial fibrosis in RCFs by increasing the Col1a1/Col3a1 ratio, thereby implicating Fmod in the pathological process of diabetic cardiomyopathy.

To further elucidate the mechanisms underlying Fmod‐induced cardiac fibrosis, we conducted transcriptome sequencing of Fmod‐overexpressing RCFs. Our findings suggest that Fmod may enhance cardiac fibrosis through diverse pathways. Integrating transcriptome data from ov‐Fmod RCFs and DCM rat hearts from our previous study revealed a consistent decrease in Col15a1 levels in both models. Col15a1, a proteoglycan predominantly synthesized by mesenchymal cells like fibroblasts, myoblasts, and adipocytes, is crucial for maintaining ECM and capillary integrity in the heart and perivascular regions [20, 21]. Col15a1‐deficient mice exhibit non‐proto‐fibrillar protein deposits in the cardiac myocardium interstitium, along with an abnormal increase in the Col1a1/Col3a1 ratio, heightened myocardial stiffness, reduced elasticity, and decreased myocardial compliance, ultimately leading to cardiomyopathy [20]. In our study, we observed a negative correlation between Fmod and Col15a1 levels in the plasma of DCM rats, indicating a potential regulatory relationship. To explore this further, we examined the mRNA expression and protein levels of Col15a1 in rat models, cell models, ov‐Fmod RCFs, and si‐Fmod RCFs. We found that Col15a1 levels decreased in both the DCM model and RCFs overexpressing Fmod, suggesting that Fmod‐mediated upregulation of the Col1a1/Col3a1 ratio in RCFs may involve the inhibition of Col15a1. Thus, our findings suggest that Fmod may promote DCM‐related myocardial fibrosis by regulating Col15a1 expression.

## Conclusions

In summary, our studies have demonstrated increased Fmod expression in both the DCM rat model and the high glucose‐induced DCM fibrotic cell model. We observed that Fmod influenced the Col1a1/Col3a1 ratio, suggesting its potential role in promoting DCM‐related cardiac fibrosis. This effect may involve the modulation of Col15a1, thereby contributing to myocardial fibrosis in DCM. However, this study has limitations, including the lack of *in vivo* validation through animal experiments and the absence of verification of upstream and downstream regulatory relationships between Fmod and Col15a1. These aspects will be addressed in future research.

## Conflict of interest

The authors declare that they have no conflicts of interest.

## Author contributions

JX and XD conceived and designed the project. XD wrote the main manuscript text and was responsible for the statistical analyses. FY and DC edited the manuscript text. XD, ZD, LY and CC were mainly responsible for experimental operations. XD and DC were responsible for the bioinformatic analyses. LY was responsible Figs 1, 2 and 4. DC was responsible for Fig. 3 and the graphical abstract. JX, FY, and DC provided the funding. All authors have reviewed the final version of the manuscript submitted for publication.

## Supporting information


**Fig. S1.** Purity testing result of SD rat primary cardiac fibroblasts.

**Table S1.** List of interference sequences.
**Table S2.** List of primer sequences.

## Data Availability

All data generated or analyzed in the course of this study have been comprehensively incorporated within this article. Any additional inquiries should be directed to the corresponding author.
