# Supplementary material for: The role of fibromodulin in myocardial fibrosis in a diabetic cardiomyopathy rat model
Source: FEBS Open Bio. 2024 Nov 26;15(3):436–46. doi: 10.1002/2211-5463.13935 (PMC11891772; doi:10.1002/2211-5463.13935)
Supplement: Supplementary file 1 — Fig. S1. Purity testing result of SD rat primary cardiac fibroblasts. [file FEB4-15-436-s001.docx]

| 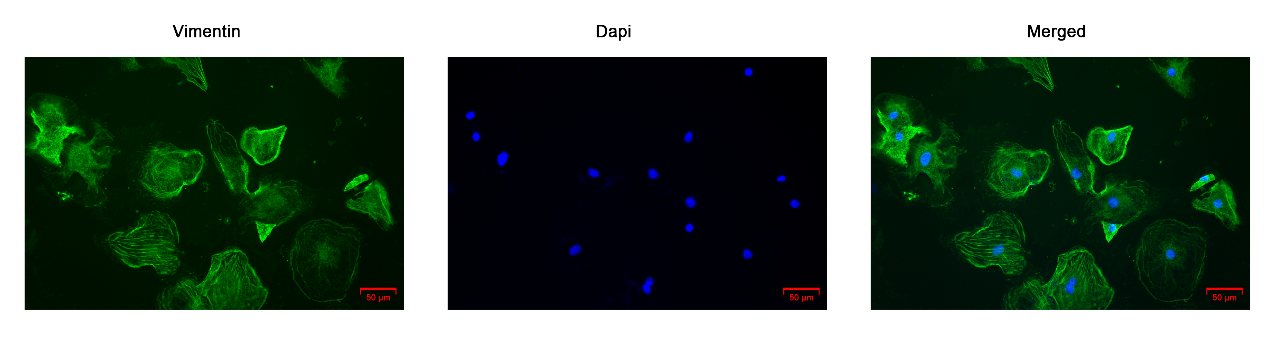 |
| --- |

**Supplementary Figure 1: Purity testing result of SD rat primary cardiac fibroblasts**

Cardiac fibroblasts from generation neonatal SD rat were isolated and inoculated for immunofluorescence identification. The immunofluorescence picture shows green for the Vimentin in the cells; blue for the nucleus of DAPI; The purity of primary cardiac fibroblasts was consistently more than 90%, as revealed by immunofluorescence analysis. The scale bar: 50 μm.
