# Supplementary material for: The role of fibromodulin in myocardial fibrosis in a diabetic cardiomyopathy rat model
Source: FEBS Open Bio. 2024 Nov 26;15(3):436–46. doi: 10.1002/2211-5463.13935 (PMC11891772; doi:10.1002/2211-5463.13935)
Supplement: Supplementary file 2 — Table S1. List of interference sequences. Table S2. List of primer sequences. [file FEB4-15-436-s002.docx]

**Table S1. The list of interference sequences**

| Name | sense (5,-3,) | antisense (5,-3,) |
| --- | --- | --- |
| siFmod | GGAGCAGCUGUACCUAGAACAdTdT | UGUUCUAGGUACAGCUGCUCCdTdT |
|  |  |  |
| siNC | UUCUCCGAACGUGUCACGUdTdT | ACGUGACACGUUCGGAGAAdTdT |

**Table S2. The list of primer sequences**

| Gene | Forward primers (5'-3') | Reverse primers (5'-3') |
| --- | --- | --- |
| Col1a1 | ATCCTGCCGATGTCGCTATCC | TTCTTGAGGTTGCCAGTCTGTTG |
| Col3a1 | GTGTGATGATGAGCCACTAGACTG | ATGACAGGAGCAGGTGTAGAAGG |
| Acta2 | AGCGTGAGATCGTCCGTGACA | CCGCCGACTCCATTCCAATGAA |
| Fmod | GGCAACCAGATTACCAGTGACAAG | GTGGTCCAAGTACAGCCTCTCC |
| Col15a1 | TGGACACAGAGGCTGGCACTT | GTGTGAAGGCATCCAGGCTGTC |
| Tubb5 | ACCAACCTACGGGGATCTGAA | TTGACTGCCAACTTGCGGA |
